# Preoperative handgrip strength is not associated with complications and health-related quality of life after surgery for colorectal cancer

**DOI:** 10.1038/s41598-020-69806-1

**Published:** 2020-08-03

**Authors:** M. van Heinsbergen, J. L. Konsten, M. J. L. Bours, N. D. Bouvy, M. P. Weijenberg, M. L. Janssen-Heijnen

**Affiliations:** 1 Department of Surgery, VieCuri Medical Centre, Tegelseweg 210, 5912 BL Venlo, The Netherlands; 2 Department of Surgery, Maastricht University Medical Centre+, P. Debyelaan 25, 6229 HX Maastricht, The Netherlands; 3 Department of Epidemiology, GROW School for Oncology and Developmental Biology, Maastricht University, P.O. Box 616, 6200 MD Maastricht, The Netherlands; 4 Department of Clinical Epidemiology, VieCuri Medical Centre, Tegelseweg 210, 5912 BL Venlo, The Netherlands

**Keywords:** Colorectal cancer, Surgical oncology

## Abstract

Colorectal cancer (CRC) treatment is associated with a high morbidity which may result in a reduced health-related quality of life (HRQoL). The pre-operative measurement of handgrip strength (HGS) might be a tool to predict the patient’s outcome after CRC surgery. The aim of this study was to evaluate the association of pre-operative HGS with the occurrence of postoperative complications and postoperative HRQoL. Stage I to III CRC patients ≥ 18 years were included at diagnosis. Demographic and clinical data as well as HGS were collected before start of treatment. HGS was classified as weak if it was below the gender-specific 25th percentile of our study population; otherwise HGS was classified as normal. The occurrence of postoperative complications within 30 days after surgery was collected from medical records. Cancer-specific HRQoL was measured 6 weeks after treatment using the EORTC QLQ-C30 and the EORTC QLQ-CR29 questionnaire. Of 295 patients who underwent surgical treatment for CRC, 67 (23%) patients had a weak HGS while 228 (77%) patients had normal HGS. 118 patients (40%) developed a postoperative complication. Complications occurred in 37% of patients with a weak HGS and in 41% of patients with a normal HGS (*p* = 0.47). After adjustment for age, sex, ASA, BMI and TNM, no significant associations between pre-operative HGS and the occurrence of postoperative complications and between HGS and HRQoL were found. We conclude that a single pre-operative HGS measurement was not associated with the occurrence of postoperative complications or post-treatment HRQoL in stage I–III CRC patients.

## Introduction

Colorectal cancer (CRC) is the second commonest cause of cancer death in the Netherlands^1^. The number of CRC patients has increased during the past years from 9,106 newly diagnosed patients in 2000 to 15,807 in 2015^2^^.^ Up to high age, the vast majority of these patients undergo surgical resection for cure or palliation^3^. Unfortunately, CRC surgery is associated with a high complication rate up to 50%, associated with a reduced health-related quality of life (HRQoL), increased hospital costs and even mortality^4,5^. Despite the introduction of laparoscopic surgery and the implementation of the Enhanced Recovery After Surgery (ERAS) protocol, certain groups with pre-existent comorbidity and a reduced functional capacity are known to be at risk for the occurrence of postoperative adverse events^6^. Preoperative risk stratification remains challenging as the tools used for the risk stratification mainly use traditional and costly examinations such as cardiac and pulmonary functioning and laboratory tests^7^. Apart from these examinations, which provide mainly anaesthesiological information on the risk of perioperative adverse events and not outcome after the end of treatment, there is a need for an easy tool to predict the patient’s outcome after colorectal surgery. Such a tool should be easy to interpret, not time- and space-consuming and cost-effective. Handgrip strength (HGS) might be a promising tool to use for this purpose, since HGS is known to be associated with nutritional status, overall body strength, aging process and functioning of the immune system in hospitalized patients^8,9^. Previous literature has shown that HGS seemed to be an effective instrument to predict postoperative complications in patients undergoing upper gastro-intestinal surgery^10^. Also, an association was found between a lower HGS and outcome measures, such as HRQoL and postoperative morbidity, in advanced cancer patients^11^. We therefore expect that HGS might be a predictor for postoperative complications as well as for certain HRQoL domains such as: global health status, physical functioning, role functioning, body image, fatigue, dyspnoea, urinary incontinence and faecal incontinence. If the measurement from such an easy applicable tool as HGS also appears to be associated with the patient’s postoperative outcome after colorectal surgery, it would enable doctors to give an adequate counselling to their patients. The results from such a tool might also be helpful to determine which patients might benefit from tailored prehabilitation programs in order to improve the patient’s preoperative functional capacity to withstand the major insult of surgery with less morbidity and better HRQoL^12,13^. Studies investigating the association between HGS and clinical and functional outcomes after CRC treatment are scarce^6,14^. In this prospective cohort study, we determined associations of pre-operative HGS with the occurrence of complications and with HRQoL in non-metastatic CRC patients six weeks after the end of treatment.

## Patients and methods

### Study design

This study is part of the Energy for life after ColoRectal cancer (EnCoRe) study, a long-term follow-up study of HRQoL issues in Dutch CRC survivors. The EnCoRe study is a prospective cohort study for stage I to III CRC patients ≥ 18 years who are diagnosed and treated in three teaching hospitals in the south-eastern part of the Netherlands: Maastricht University Medical Center+, VieCuri Medical Centre, and Zuyderland Medical Center. The presence of stage IV CRC, inability to understand the Dutch language or comorbidities that may obstruct successful participation, including cognitive disorders (e.g. Alzheimer disease) and severe visibility or hearing disorders, were reasons to exclude patients. Patient recruitment started in April 2012 and is ongoing^15^. The EnCoRe study has been approved by the Medical Ethics Committee of the University Hospital Maastricht and Maastricht University, the Netherlands, and informed consent is obtained from all participants. All methods were performed in accordance with the relevant guidelines and regulations. In the EnCoRe study, longitudinal measurements are performed at diagnosis (pre-treatment) and at 6 weeks, 6 months, and 1, 2 and 5 years after the end of CRC treatment. For the present analyses, only data from the pre-treatment and 6-week post-treatment time points have been used, collected during the first 4.5 years of patient inclusion and follow-up of the EnCoRe study (until November 2016).

### Data collection

Demographic and clinical data are being collected from medical records by trained nurse practitioners and patient visits were done by research dieticians. These data include information on gender, age at diagnosis, marital status, body mass index (BMI), malnutrition universal screening tool (MUST), (neo-)adjuvant therapy, postoperative tumour stage (TNM stage I, II, III), surgical procedure, surgical technique (laparoscopic vs. open), the occurrence of postoperative complications within 30 days after surgery (yes, no), type of complication (cardiopulmonary, infectious, surgical and other), number of readmissions within 30 days after surgery, length of hospital stay, and ASA-classification (1–6).

### Handgrip strength

HGS was measured (in kg) before start of oncological treatment using a hand-held dynamometer. Patients were seated in a chair with their elbow flexed at 90º and the forearm in the neutral position. Two HGS measurements were performed using the dominant hand (with sufficient rest in-between measurements), of which the highest value was recorded as the patient’s maximum HGS^16^. For statistical analyses, HGS was used as a continuous as well as a categorised variable. Patients were categorized as having weak or normal HGS. HGS was classified as weak if it was below the gender-specific 25th percentile of our study population; otherwise HGS was classified as normal. In the current database, cut-off values for weak versus normal HGS were 38 kg for male patients and 21 kg for female patients.

### Patient reported outcome measures

Cancer-specific HRQoL was measured 6 weeks after treatment using selected domains of the valid and reliable European Organisation for Research and Treatment of Cancer Quality of Life Questionnaire-Core 30 (EORTC QLQ-C30, version 3.0), complemented with the CRC-specific CR29 module (v2.1). The EORTC QLQ-C30 questionnaire consists of 30 questions and includes subscales on global QoL, on physical, role, social, emotional, and cognitive functioning, and on specific symptoms^17^. The EORTC QLQ-CR29 questionnaire is designed specifically for CRC patients, and consists of 29 items addressing gastrointestinal symptoms, chemotherapy side effects, stoma-related problems, defecation problems, pain and problems with micturition, and separate items addressing sexual function for men and women^18^. For both questionnaires, subscale scores range from 0 to 100 points, with a high score on the functioning subscales representing a high level of functioning and a high score on the symptom subscales representing a high level of symptoms. We preselected certain subscales of the EORTC questionnaires for the present analyses, based on clinical relevance. Within the subscales, global QoL, physical functioning, role functioning, and body image were considered to have a potential association with HGS, while fatigue, dyspnoea, urinary incontinence and faecal incontinence were considered as clinical relevant symptom subscales.

### Statistical analyses

Patient characteristics were summarized with descriptive statistics. Patient, tumour and treatment characteristics possibly associated with the postoperative complication rate were first tested in univariate analyses performed using tests or Fisher’s exact tests. In case of associations (*p* < 0.10) between these characteristics and the postoperative complication rate in univariate analysis, these variables were included as possible confounders in the multivariable logistic regression analysis to derive a final confounder-adjusted model of the independent association between preoperative HGS and the presence of postoperative complication(s). Based on theoretical considerations also sex, age, BMI and TNM stage were added as possible confounders to the multivariable analysis^19–21^. To add or remove a variable from the model, the corresponding P-value had to be smaller than 0.10 and greater than 0.10, respectively, in a backward stepwise analysis. In order to guarantee both clinical applicability and statistical validity, separate multivariable analyses were performed with HGS as a categorical or as a continuous independent variable included in the regression models.

Mean HRQoL scores were compared between HGS categories using independent sample T-tests in case of a normal distribution or Mann–Whitney U tests for variables without a normal distribution. Variables that were associated with domains of HRQoL in univariate analyses (*p* < 0.10) as well as sex, age, BMI and TNM stage were included in a multivariable linear regression analysis to derive a final model of the confounder-adjusted independent association between preoperative HGS and certain domains of HRQoL^20,22,23^. To add or remove a variable from the backward stepwise model, the corresponding P-value had to be smaller than 0.10 and greater than 0.10, respectively. Values of *p* < 0.05 were considered statistically significant. All statistical analyses were performed using IBM® SPSS® Statistics, version 24.

## Results

**Figure 1:**
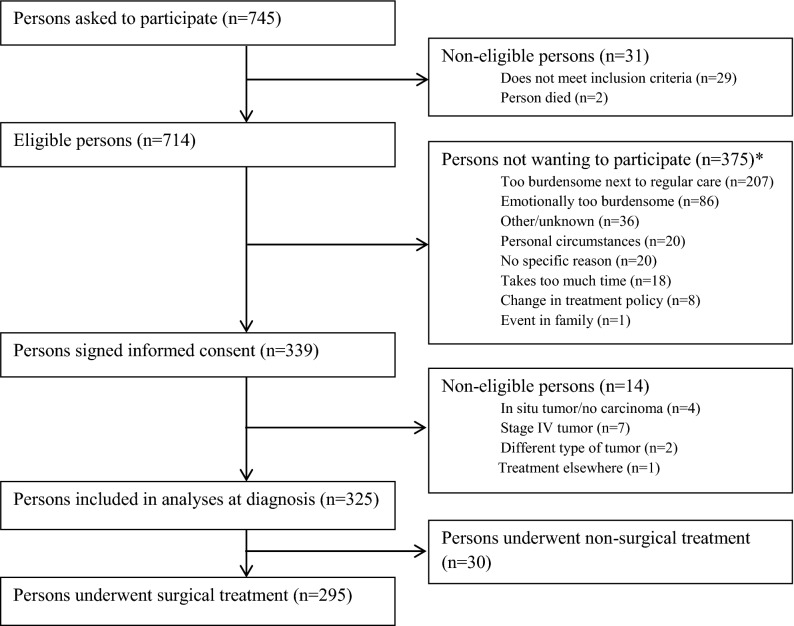
Flow-diagram of inclusion of individuals within the EnCoRe study and included in the analyses presented in this paper. Data of home visits performed before Nov. 1st 2016 were included in the analyses. *Totals do not add up because some individuals reported multiple reasons for non-participation.

A total of 745 patients were asked to participate in the study, of whom 31 patients were not eligible for inclusion. Of the 714 eligible patients, 339 patients wanted to participate in the study and signed the informed consent form. After signing the informed consent form another 14 patients had to be excluded at moment of diagnosis. A total of 325 patients were enrolled of whom 295 patients (91%) underwent surgical treatment for CRC between April 2012 and November 2016. **(**Fig. 1**)** The overall mean age at diagnosis was 67 years (range 36–88) and 218 patients (66%) were male.

Of the 295 patients who underwent surgical treatment for CRC, 67 (23%) patients were categorised as having a weak HGS while 228 (77%) patients had a normal HGS. Mean HGS was 45 (SD = 10) kilograms among men and 26 (SD = 7) kilograms among women. At inclusion, baseline characteristics of patients with either a weak or a normal HGS were similar, except for age which was significantly higher in patients with a weak HGS (*p* < 0.01). Patients with a weak HGS also seemed to be diagnosed with a higher stage of ASA-classification and a higher risk of malnutrition, although both did not reach statistical significance (*p* = 0.085 and *p* = 0.065, respectively) **(**Table 1**)**.

**Table 1:** Comparison of patient baseline characteristics between HGS groups of patients who underwent surgical treatment for colorectal cancer.

	Weak HGS (n = 67)	Normal HGS (n = 228)	*p* value
**Median age in years (range)**	74 (55–88)	66 (36–85)	< 0.01
**Gender**			
Male	43 (64.2%)	152 (66.7%)	0.769
Female	24 (35.8%)	76 (33.3%)	
**TNM staging**			
Stage I	16 (23.9%)	70 (30.8%)	0.543
Stage II	17 (25.4%)	51 (22.5%)	
Stage III	34 (50.7%)	106 (46.7%)	
**Surgical technique**			
Open	16 (23.9%)	46 (20.2%)	0.323
Laparoscopic	49 (73.1%)	180 (78.9%)	
Transanal endoscopic	2 (3.0%)	2 (0.9%)	
**Tumour location**			
Right/transverse/left colon	28 (41.8%)	78 (34.2%)	0.334
Sigmoid/rectum	39 (58.2%)	150 (65.8%)	
**ASA**			
Grade I	7 (10.4%)	60 (26.3%)	0.009
Grade II	44 (65.7%)	134 (58.8%)	
Grade III	15 (22.3%)	34 (14.9%)	
Grade IV	1 (1.5%)	0 (0%)	
**BMI (kg/m**^**2**^**)**			
< 25	23 (34.4%)	6 (27.6%)	0.518
25.1–30	24 (35.8%)	98 (43.0%)	
> 30.1	13 (19.4%)	55 (24.1%)	
Unknown*	7 (10.4%)	12 (5.3%)	
**MUST score**			
Low risk	51 (76.1%)	190 (83.3%)	0.098
Medium risk	4 (6.0%)	18 (7.9%)	
High risk	5 (7.5%)	4 (1.8%)	
Unknown*	7 (10.4%)	11 (7.0%)	
**Neo-adjuvant treatment**			
None	54 (80.6%)	171 (75.0%)	0.320
Radiotherapy	6 (9.0%)	16 (7.0%)	
Chemoradiotherapy	7 (10.4%)	41 (18.0%)	
**Adjuvant treatment**			
None	49 (73.1%)	149 (65.4%)	0.316
Radiotherapy*	0 (0%)	1 (0.4%)	
Chemotherapy	18 (26.9%)	78 (34.2%)	
**Readmission within 30d after surgery**			
Yes	6 (9.0%)	34 (14.9%)	0.309
No	61 (91.0%)	194 (85.1%)	
**Length of hospital stay (days)**			
Mean	9.05	7.83	0.204
Median	6	6	
Range	1–82	3–45	

*ASA* American Society of Anaesthesiologists, *BMI* body mass index, *MUST* malnutrition universal screening tool.

*These values were excluded for univariate and multivariate analysis.

### Postoperative complications

Out of the 295 patients who underwent colorectal surgery, 118 patients (40%) developed a postoperative complication; 37% among patients with a weak HGS at baseline and 41% among those with a normal HGS at baseline (*p* = 0.465). Within these 118 patients, 201 complication events were observed: 33 (16%) cardiopulmonary, 25 (12%) infectious, 88 (44%) surgical and 55 (27%) other complication events, which did not differ significantly between patients with weak or normal HGS (Table 2). The median length of hospital stay was 6 days (range 1–82 days) for both weak and normal HGS (*p* = 0.204). Among patients with a weak HGS, 6 patients (9%) were readmitted to the hospital within 30 days after treatment versus 34 patients (15%) among the patients with a normal HGS (*p* = 0.309) (Table 1). Also after adjustment for age, sex, ASA, BMI and TNM stage as confounders in multivariable analyses, no significant associations were found between HGS and the occurrence of postoperative complications (HGS categorized as weak versus normal: OR 0.72; 95% CI 0.38–1.39; HGS as continuous variable per kg: OR 1.00; 95% CI 0.97–1.03).

**Table 2:** Univariate analysis for associations between handgrip strength (HGS) as a categorical variable and the occurrence of postoperative complications within 30 days.

	Weak HGS	Normal HGS	*p* value
**Complication within 30 days after treatment**	**N (%)**	**N (%)**	
Patients	25 (37)	93 (41)	0.465
Events	39	162	
**Type of postoperative complication**	**N events (%)**	**N events (%)**	
Cardiopulmonary	7 (18)	26 (16)	0.693
Infectious*	4 (10)	21 (13)	0.475
Surgical	16 (41)	72 (44)	0.171
Other	12 (31)	43 (27)	0.798

*Infectious complications other than surgical or pulmonary.

### Quality of life

In univariate analyses, a weak pre-operative HGS was associated with worse physical functioning and more faecal incontinence postoperative. On average, patients with normal HGS scored 7.68 points higher on physical functioning than patients with weak HGS (B = 7.68; 95% CI 1.52–13.83), and patients with normal HGS scored on average 9.3 points lower on faecal incontinence (B = −9.30; 95% CI − 17.38 to − 1.22). After adjustment for age, sex, ASA, BMI and TNM, only the association between HGS and faecal incontinence remained statistically significant (B = −9.50; 95% CI − 18.32 to − 0.68). No significant associations were found between HGS and global QoL, role functioning, body image, fatigue, dyspnoea, and urinary incontinence (Table 3).

**Table 3:** Univariate and multivariable linear regression analysis for associations between the pre-operative handgrip strength (HGS) and health-related quality of life (HRQoL) domains.

	Mean score	Pre-operative HGS (categorical)				Pre-operative HGS (continuous)			
		Unadjusted B (95% CI)	*p* value	Adjusted B (95% CI)	*p* value	Unadjusted B /1 kg (95% CI)	*p* value	Adjusted B /1 kg (95% CI)	*p* value
**Functional subscales**									
Global Health Status	73.47	− 0.47 (− 6.38 to 5.44)	0.875	0.94 (− 5.46 to 7.34)	0.773	− 0.11 (− 0.31 to 0.09)	0.280	− 0.15 (− 0.48 to 0.18)	0.358
Physical Functioning	75.46	7.68 (1.52 to 13.83)	0.015	5.06 (− 1.15 to 11.26)	0.110	0.31 (0.11 to 0.52)	0.003	0.07 (− 0.25 to 0.39)	0.662
Role Functioning	68.46	− 3.27 (− 12.23 to 5.69)	0.472	− 1.35 (− 10.99 to 8.30)	0.783	− 0.08 (− 0.39 to 0.22)	0.585	− 0.23 (− 0.72 to 0.27)	0.365
Body Image	84.73	− 3.93 (− 10.970 to 3.11)	0.279	− 0.36 (− 7.86 to 7.15)	0.926	− 0.18 (− 0.42 to 0.06)	0.133	− 0.30 (− 0.68 to 0.08)	0.122
**Symptom subscales**									
Fatigue	30.02	0.31 (− 7.03 to 7.66)	0.933	− 0.34 (− 8.22 to 7.55)	0.933	− 0.09 (− 0.34 to 0.16)	0.468	− 0.05 (− 0.45 to 0.36)	0.812
Dyspnoea	13.42	− 1.11 (− 8.89 to 6.67)	0.779	1.72 (− 6.27 to 9.71)	0.672	− 0.19 (− 0.45 to 0.07)	0.157	− 0.11 (− 0.52 to 0.30)	0.602
Urinary Incontinence	8.41	− 1.19 (− 7.05 to 4.67)	0.688	0.63 (− 5.75 to 7.01)	0.845	− 0.18 (− 0.38 to 0.01)	0.067	0.13 (− 0.20 to 0.46)	0.428
Faecal Incontinence	14.16	− 9.30 (− 17.38 to − 1.23)	0.024	− 9.50 (− 18.32 to − 0.68)	0.035	− 0.19 (− 0.47 to 0.08)	0.166	0.11 (− 0.33 to 0.55)	0.616

The multivariable analyses were performed with HGS as a categorical (normal HGS group as reference category) as well as a continuous variable. Age, sex, American Society of Anaesthesiologists (ASA) score, body mass index (BMI) and TNM stage were included as confounders in the multivariable analyses.

In univariate analyses where HGS was included as a continuous variable, each kilogram increase in HGS was associated with a 0.31 points higher physical functioning score (B = 0.31; 95% CI 0.11–0.52). After adjustment for age, sex, ASA, BMI and TNM stage, no significant associations between HGS and any of the investigated HRQoL domains were found (Table 3).

### Transanal endoscopic microsurgery

A total of 4 included patients underwent a transanal endoscopic microsurgical (TEM) resection. Although TEM-treated patients are a selected group of patients that may differ in comorbidity and prognosis from the other included patients, exclusion of these patients in a sub analysis did not alter our main results.

## Discussion

In our study among 295 patients who underwent surgical treatment for CRC, no significant associations of pre-operative HGS with the occurrence of complications and with HRQoL after CRC treatment were found. The fact that no association between a weak pre-operative HGS and the occurrence of complications after colorectal surgery was found in our study is in contrast to a previous study that showed an association between a lower pre-operative HGS and the occurrence of postoperative complications after adjustment for age and gender, within 30 days after abdominal surgery^10,24^. However, these studies included patients who underwent major surgery because of oesophageal or gastric cancer. This type of surgery is known as high-risk surgery in patients with more frailty than our patients, with an even higher morbidity rate compared to our study population^25^. Patients of the current study might consist of a relatively healthier and less frail study population, partly because of exclusion of patients with stage IV tumours. Besides that, there is a possibility that the patients’ better or poorer physical function affected their willingness of participation. This might be a possible explanation for the discrepancy between our results and the previously mentioned studies^10,24^. Nevertheless, a recent Dutch study among a similar population of CRC patients published results in line with our findings, as no significant association between pre-operative HGS as a single measurement and the occurrence of postoperative complications was found^6^. On the other hand, the same Dutch study demonstrated a significant association between an overall better pre-operative physical fitness, defined by a combination of physical tests, and a lower incidence of postoperative complications^6^. The absence of an association between HGS as a single measurement and the incidence of postoperative complications is in line with the results of our study as well as results from other recent studies that found no association between a pre-operative HGS as a single functional performance measurement, and the occurrence of postoperative complications^26,27^.

For the association between HGS and HRQoL, a previous cross-sectional study showed a significant association between lower HGS and lower global QoL in frail elderly people^28^. In prospective studies among patients with breast cancer and advanced small non-cell lung carcinoma, a lower HGS before treatment was also related to lower HRQoL thereafter^29,30^. Studies investigating a similar direct association between HGS and patients’ HRQoL in CRC survivors are lacking. For CRC patients we only know from cross-sectional studies that there is a significant association between a low HGS and the presence of sarcopenia after treatment^31^. Sarcopenia is often present in CRC patients and related to a lower HRQoL with worse scores for physical functioning and increased fatigue^32^. Nevertheless, in our study an association between HGS and HRQoL could not be found after adjustment for differences in age, sex, ASA, BMI and TNM stage. Previous studies have shown that cancer treatment-related effects that alter muscle strength and body composition (either directly or indirectly via physical inactivity) promote loss of physical function, which further exacerbates fatigue and HRQoL^33^. This could be a reasonable explanation for the fact that the use of a single pre-treatment HGS measurement, as we investigated in our study, does not seem to be associated to postoperative outcome in terms of HRQoL after adjustment for other potential determinants of HRQoL.

It seems that a low HGS alone cannot be used as a measure of increased vulnerability towards stressors which can result in a higher complication rate and a lower HRQoL. This state of increased vulnerability towards stressors is called frailty^34^. Although HGS correlates to patient’s overall muscle strength, bone density, nutritional status and frailty even better than chronological age, the use of HGS as a single measurement of the patients’ physical capacity could be a limitation of our study^14^. For this paper we consciously opted to use a single HGS measurement as it could possibly be a simple, not time- and space-consuming and cost-effective indicator, which has previously been shown to have an association with postoperative complications and HRQoL in other surgical populations^10,24,30^.

Future research should focus on the association between different physical and functional parameters such as weight loss, physical activity and HGS as tools for the patients’ risk stratification, and post-treatment morbidity as well as the patients’ HRQoL^34^. Identifying a deficit in one of these parameters before start of the treatment can be of great additional value as we know that the enhancement of certain parameters can lead to an improvement of the patients’ functional capacity which enables them to withstand an incoming stressor^35^. This process of enhancing the patients’ preoperative physical functioning has been termed prehabilitation and has proven to result in better postoperative outcomes in CRC patients^13,36^.

The strength of our study lies in its prospective design with adequate sample size, the use of HGS as an objective physical measurement and of the EORTC questionnaires as a validated HRQoL measurement. On the one hand, the absence of a baseline HRQoL measurement could be a limitation of our study; on the other hand, the reliability of such a preoperative measurement might be doubtful as it can be biased by the fact that these patients just received a diagnosis of cancer. Another limitation could be the occurrence of selection since younger patients were probably more willing to participate in our study, which could have led to a healthier and less frail study population and thus a lower complication rate and a better HRQoL^37^. However, this is a prospective study with very low loss to follow-up between baseline and follow-up measurements (0.7% for the postoperative complication measurement and 4.4% for the HRQoL measurement), therefore it is unlikely that a potentially fitter baseline population has biased the investigated associations.

Based on the results of our study, we can conclude that a single pre-operative HGS measurement was not associated with postoperative complication rate and post-treatment HRQoL. Future research should focus on other physical and functional tests and their associations with postoperative morbidity. The identification of such tests enables clinicians to determine which patients might benefit from tailored prehabilitation programs in order to improve the patient’s functional capacity, which might lead to less treatment complications and a better QoL.
